# Evaluation of confounding in epidemiologic studies assessing alcohol consumption on the risk of ischemic heart disease

**DOI:** 10.1186/s12874-020-0914-6

**Published:** 2020-03-14

**Authors:** Joshua D. Wallach, Stylianos Serghiou, Lingzhi Chu, Alexander C. Egilman, Vasilis Vasiliou, Joseph S. Ross, John P. A. Ioannidis

**Affiliations:** 1 Department of Environmental Health Sciences, Yale School of Public Health, 60 College Street, 4th Floor, Room 411, New Haven, CT 06510 USA; 2 Collaboration for Research Integrity and Transparency (CRIT), Yale School of Medicine, New Haven, CT 06510 USA; 3 Center for Outcomes Research and Evaluation, Yale-New Haven Health System, New Haven, CT 06510 USA; 4 Meta-Research Innovation Center at Stanford (METRICS), Stanford School of Medicine, 1265 Welch Rd, MSOB X306, Stanford, CA 94305 USA; 5 Department of Epidemiology & Population Health, Stanford School of Medicine, 150 Governor’s Lane, Stanford, CA 94305 USA; 6 Section of General Medicine, Department of Internal Medicine, Yale School of Medicine, 367 Cedar Street, Ste 405B, New Haven, CT 06510 USA; 7 National Clinician Scholars Program, Yale School of Medicine, 367 Cedar Street, Ste 405B, New Haven, CT 06510 USA; 8 Department of Health Policy and Management, Yale School of Public Health, 60 College Street, New Haven, CT 06510 USA; 9 Stanford Prevention Research Center, Department of Medicine, Stanford University School of Medicine, 1265 Welch Rd, MSOB X306, Stanford, CA 94305 USA; 10 Department of Statistics, Stanford University School of Humanities and Sciences, Stanford, CA 94305 USA

**Keywords:** Confounding, Bias, Observational studies, Alcohol exposure, Ischemic heart disease, Adjustment

## Abstract

**Background:**

Among different investigators studying the same exposures and outcomes, there may be a lack of consensus about potential confounders that should be considered as matching, adjustment, or stratification variables in observational studies. Concerns have been raised that confounding factors may affect the results obtained for the alcohol-ischemic heart disease relationship, as well as their consistency and reproducibility across different studies. Therefore, we assessed how confounders are defined, operationalized, and discussed across individual studies evaluating the impact of alcohol on ischemic heart disease risk.

**Methods:**

For observational studies included in a recent alcohol-ischemic heart disease meta-analysis, we identified all variables adjusted, matched, or stratified for in the largest reported multivariate model (i.e. potential confounders). We recorded how the variables were measured and grouped them into higher-level confounder domains. Abstracts and Discussion sections were then assessed to determine whether authors considered confounding when interpreting their study findings.

**Results:**

85 of 87 (97.7%) studies reported multivariate analyses for an alcohol-ischemic heart disease relationship. The most common higher-level confounder domains included were smoking (79, 92.9%), age (74, 87.1%), and BMI, height, and/or weight (57, 67.1%). However, no two models adjusted, matched, or stratified for the same higher-level confounder domains. Most (74/87, 85.1%) articles mentioned or alluded to “confounding” in their Abstract or Discussion sections, but only one stated that their main findings were likely to be affected by residual confounding. There were five (5/87, 5.7%) authors that explicitly asked for caution when interpreting results.

**Conclusion:**

There is large variation in the confounders considered across observational studies evaluating the impact of alcohol on ischemic heart disease risk and almost all studies spuriously ignore or eventually dismiss confounding in their conclusions. Given that study results and interpretations may be affected by the mix of potential confounders included within multivariate models, efforts are necessary to standardize approaches for selecting and accounting for confounders in observational studies.

## Background

Over the past few years, there have been a growing number of studies outlining both harmful and potentially protective effects of alcohol consumption on the risk of various health-related outcomes, including ischemic heart disease [1–7]. Many of these studies, which have gained widespread attention in the media [8, 9], have the potential to influence consumer behavior and can create uncertainty and false notions regarding healthy or unhealthy practices [10, 11]. The burden of disease from alcohol is undoubtedly high [8], but there is less clarity about estimates of risk (or even protection) with low levels of consumption [6–8, 12, 13]. Typically, the reported associations between alcohol and health-related outcomes come from observational studies, which have inherent methodological limitations that generate bias and confounding [14, 15].

Confounding is the bias resulting from the presence of common causes of exposures and outcomes [14, 16, 17]; thus confounders can distort observed exposure-outcome associations [14, 18, 19]. Although there are numerous techniques that can be used to account for confounding in observational research, it is very challenging to completely exclude the impact of unmeasured residual confounding [17]. Furthermore, many potential confounders may be unknown to researchers and can be difficult to identify or measure. Among different investigators studying the same exposures and outcomes, there may be a lack of consensus about potential confounding variables that should be considered as matching (i.e. the selection of comparators or comparison groups with respect to one or more potential confounders), adjustment (i.e. the inclusion of potential confounders in multivariate analyses), or stratification variables (i.e. the fixing of levels of confounders by producing groups (strata) within which confounders do not vary and evaluating associations within stratum of the confounder(s)). As a result, individual studies may evaluate different confounders and/or report on certain subsets of a larger pool of potential variables in published articles.

Recently, the Global Burden or Disease (GBD) 2016 Alcohol Collaborators published a meticulous systematic analysis of alcohol burden across the world, which included separate meta-analyses for 23 health outcomes [8]. Ischemic heart disease was the only outcome with significant evidence for a J-shaped curve [8], supporting previous claims that lower-volume alcohol intake may be associated with no harm or even protective effects [9, 20, 21]. However, all of these results come from observational studies, where confounders may contribute to both favorable or unfavorable associations [22]. For instance, it has been proposed that some or all of the U-or-J-shaped dose-response trends may be attributable to unmeasurable characteristics that are associated with alcohol consumption and cardiovascular outcomes [13, 22]. While some meta-analyses have suggested that adjusting for common confounders, such as smoking, age, and sex, does not alter the observed effect estimates [9], others claim that individual studies with adjusted effect estimates have lower (attenuated) protective effects [20].

Concerns have been raised that confounding factors may affect the results obtained for the alcohol-ischemic heart disease relationship, as well as their consistency and reproducibility across different studies [21]. Therefore, we systematically assessed whether individual observational studies evaluating the impact of alcohol on ischemic heart disease considered the same, similar, or different confounders and how much heterogeneity existed on how these confounders were defined and operationalized in matching, stratifying, or adjusting for them in the analyses. Additionally, we examined how authors of the individual studies considered confounding bias when interpreting their findings.

## Methods

### Design

#### Data identification and eligibility

We evaluated the individual studies included in the ischemic heart disease meta-analysis conducted by the GBD 2016 Alcohol Collaborators [8]. We did not perform a separate systematic search because the GBD meta-analysis is recent and comprehensive. Briefly, the GBD authors performed a systematic review of the literature published between 1 January 1950 and 31 December 2016 using PubMed, the Global Health Data Exchange, and the references of previous meta-analyses. Studies were excluded if they: did not report on the association between alcohol use and ischemic heart disease; were not cohort, case-control, or case-crossover studies; did not report a relative measure of risk or cases and non-cases among the exposed and un-exposed; did not report dose-response amounts of alcohol use; and did not have study endpoints that met the case definition used in the GBD 2016 report [8].

#### Study characteristics

One author (JDW) manually screened all studies included in the ischemic heart disease meta-analyses performed by the GBD 2016 collaborators, and excluded articles that did not report any information about bivariate or multivariate analyses for an alcohol-ischemic heart disease relationship. For all eligible articles, we then recorded: the first author’s name; year of publication; study design (i.e. case-control or cohort); study location (i.e. North America, Europe, Asia, or Other), overall sample size, and name of the journal publishing the study. InCites™ Journal Citation Reports (JCR) was used to determine the 2017 JCR impact factor for each journal. As in previous evaluations, we recorded the most recent impact factor for each journal for consistency, despite the different publication dates of the eligible articles [23–25].

#### Confounding variables

For all eligible articles, we screened the Methods and Results sections to identify the adjustment variables (i.e. potential confounders) included in the multivariable models analyzing the impact of alcohol exposure on ischemic heart disease. We recorded how the adjustment variables were measured (e.g. “age continuous” vs. “age categorical”) as well as their levels (e.g. age categorical: < 50, > 50 years). In studies with two or more multivariate models (e.g. a small model adjusted for age vs. a larger model adjusting for all statistically significant factors), we extracted the data from the largest model. We then recorded which variables were used as matching and stratification variables, but were not included as covariates in the multivariable models. However, with the exception of gender, we did not capture whether the analyses were restricted to certain values of specific variables, e.g. based on eligibility criteria. Lastly, all potential variables were then grouped into higher-level confounder domains (e.g. “age continuous” and “age categorical” into “age”).

#### Confounding statements and bias consideration

Following the same protocol as a previous evaluation [26], we screened the Abstract and Discussion sections of the included studies using six standardized pre-specified questions concerning confounding statements and bias consideration (Table 1).

**Table 1:** Assessment of consideration of confounding bias in Abstracts and Discussion [26]

1. “Do the authors mention confounding using explicitly the terms “confounder(s),” “confounding,” “confound,” or do they allude to it without using those terms, or is confounding not considered at all?” [26]	
2. “Do the authors mention bias using explicitly the term “bias”?” [26]	
3. “Do the authors mention specific confounders that have not been adjusted for? (If yes, what were the reasons? If not, were there unspecified unmeasured confounders without specifically stating which ones?” [26]	
4. “Do the authors state that their main findings are likely, possibly, or unlikely affected by residual confounding?” [26]	
5. “Do the authors state that their findings need to be interpreted with caution due to confounding?” [26]	
6. “Do the authors call for caution or indicate limitations or uncertainty due to possible confounding or other bias in their conclusions?” [26]	

### Analysis

Descriptive statistics were used to characterize eligible articles and their consideration of confounding variables within our higher-level domain categories. Separate “data microarrays” were created to illustrate the confounders that were adjusted for by each article [27]. Figures were created for higher-level confounder domains and articles were ordered in descending order based on the number of confounders considered within studies (x-axis) and times each confounder was considered across studies (y-axis). The figures were color coded to indicate whether each study adjusted, stratified, or matched for each confounder and whether each confounder was considered as a continuous or categorical variable. As suggested during peer review, we also examined the proportion of articles with confounding statements and bias consideration stratified by publication date (before 1990, 1990–1999, 2000–2009, 2010+). All analyses were conducted in R.

## Results

### Study description

Among the 93 articles referenced by the GBD ischemic heart disease meta-analysis, six were excluded because they did not meet the selection criteria (duplicate (*n* = 2), non-English language (*n* = 1), could not be located by a librarian (*n* = 1), and did not explicitly report results from analyses evaluating the impact of alcohol on ischemic heart disease (*n* = 2)). Of the 87 remaining eligible articles (Additional file 1: Table S1), 78 were published in a journal with a 2017 JCR impact factor (median 6.1 (interquartile range [IQR], 4.2–18.9)) (Table 2).

**Table 2:** Characteristics of 87 observational studies evaluating the impact of alcohol consumption on ischemic heart disease

Study characteristics	No. (%)Median (Interquartile Range)		
	Cohort	Case-control	Total
Number of studies	70	17	87
Publication year			
*< 1990*	8 (11.4)	2 (11.8)	10 (11.5)
*1990–1999*	26 (37.1)	5 (29.4)	31 (35.6)
*2000–2009*	25 (35.7)	8 (47.1)	33 (37.9)
*2010+*	11 (15.7)	2 (11.8)	13 (14.9)
Location			
*North America*	33 (47.1)	4 (23.5)	37 (42.5)
*Europe*	24 (34.3)	9 (52.9)	33 (37.9)
*Asia*	9 (12.9)	1 (5.9)	10 (11.5)
*Other*	4 (5.7)	3 (17.7)	7 (8.1)
Population			
*All*	30 (42.9)	11 (64.7)	41 (47.1)
*Males only*	33 (47.1)	3 (17.7)	36 (41.4)
*Females only*	7 (10.0)	3 (17.7)	10 (11.5)
Sample size	11,957 (4843–49,566)	1602 (899–2710)	7735 (2634–36,191)

There were 70 (70 of 87, 80.5%) cohort and 17 (17 of 87, 19.5%) case-control studies (Table 2), which included a median of 11,957 (IQR, 4843–49,566) and 1602 (IQR, 899–2710) participants, respectively. The majority of the studies were conducted in either North America (37, 42.5%) or Europe (33, 37.9%). Nearly half of the studies included both males and females (41, 47.1%). Two articles did not report results from multivariate regression analyses.

### Confounders considered

**Fig. 1:**
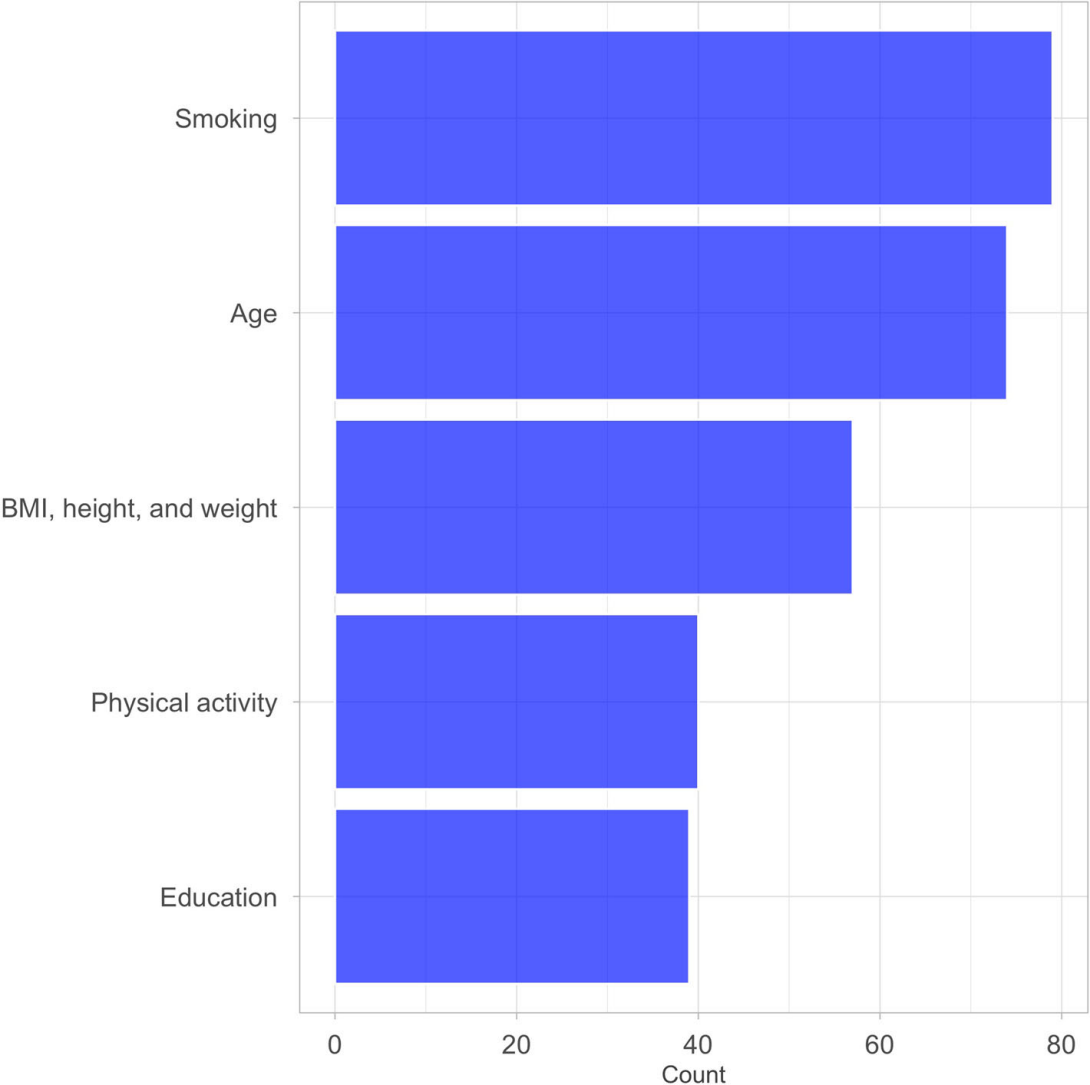
The most common higher-level confounder domains considered in 85 observational studies on alcohol and ischemic heart disease risk. Refer to Additional file 2: Figure S1 for a larger data microarray

**Fig. 2:**
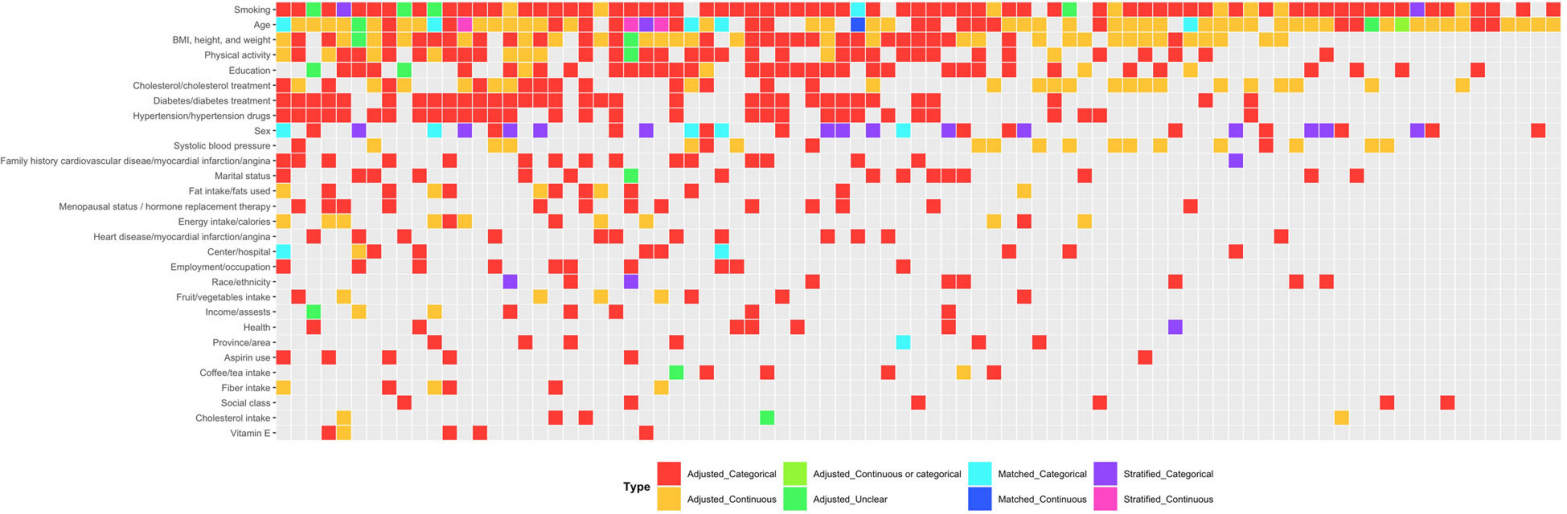
A “data microarray” illustrating the higher-level confounder domains considered in 85 observational studies on alcohol and ischemic heart disease risk. Domains are ordered based on how many times they were included in multivariate models. Colors represent whether domains were adjustment, stratification, or matching variables and how they were measured. Refer to Additional file 3: Figure S2 for a larger data microarray

The largest models in the 85 articles conducting multivariate regression analyses included a median of 9 (IQR, 5–12) adjustment, stratification, and/or matching variables. The vast majority of the 760 total variables were adjustment variables (716, 94.2%); 27 (3.6%) were stratification variables and 17 (2.2%) were matching variables in case-control studies. The 760 variables could be divided into 88 higher-level confounder domains (e.g., “history of angina” and “myocardial infarction” as “heart disease/myocardial infarction/angina”) (Additional file 2: Figure S1). The five most commonly considered higher-level domains in the 85 articles were smoking (79, 92.9%), age (74, 87.1%), BMI, height, and/or weight (57, 67.1%), physical activity (40, 47.1%), and education (39, 45.9%) (Fig. 1). A total of 33 higher-level domains were only included in one article (Additional file 2: Figure S1). No two articles evaluating the impact of alcohol on ischemic heart disease adjusted, matched, or stratified for the exact same higher-level confounder domains (Fig. 2, Additional file 3: Figure S2).

**Fig. 3:**
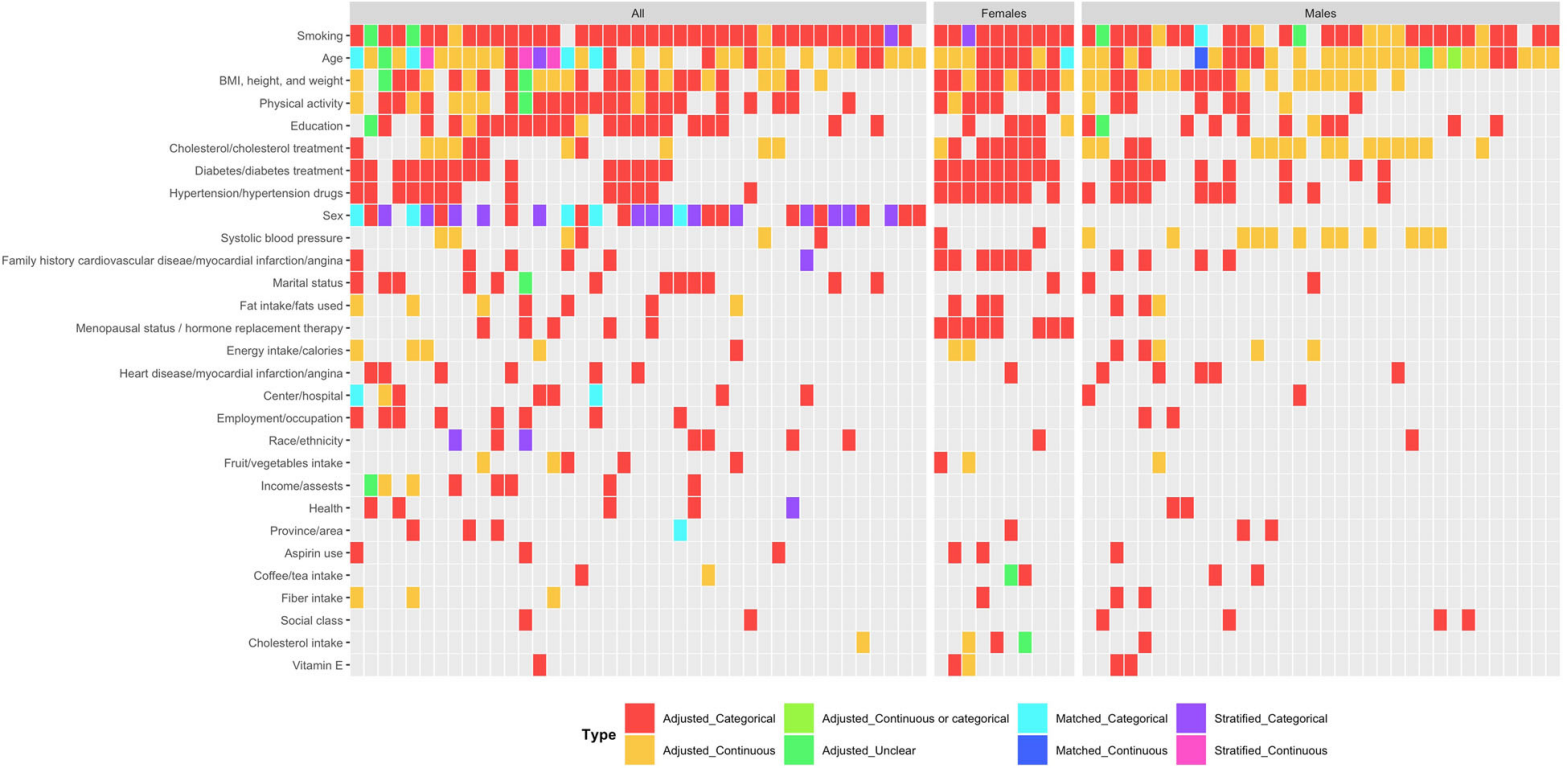
A “data microarray” illustrating the higher-level confounder domains considered in 85 observational studies on alcohol exposure and ischemic heart disease, stratified by the type of population considered. Domains are ordered based on how many times they were included in multivariate models. Colors represent whether domains were adjustment, stratification, or matching variables and how they were measured. Refer to Additional file 4: Figure S3 for a larger data microarray

The 41 articles evaluating both males and females included a total of 391 variables, which could be divided into 65 higher-level confounder domains. The five most commonly considered higher-level domains in the 41 articles were smoking (39, 95.1%), age (35, 85.4%), sex (31, 75.6%), BMI, height, and/or weight (27, 65.9%), and education (23, 56.1%) (Fig. 3, Additional file 4: Figure S3). When limited to the 10 articles evaluating only female participants, there were 126 total variables, which could be divided into 37 higher-level confounder domains. The five most common domains were smoking (10, 100.0%), age (10, 100.0%), BMI, height, and/or weight (10, 100.0%), diabetes and diabetes treatment (9, 90.0%), and hypertensions and hypertension drugs (8, 80.0%). The 34 articles with only male participants contained 243 variables, which could be divided into 58 higher-level confounder domains. The five most common domains in the 34 articles were smoking (30, 88.2%), age (29, 85.3%), BMI, height, and/or weight (20, 58.8%), cholesterol/cholesterol treatment (16, 47.1), and systolic blood pressure (12, 35.3%).

Among the 74 articles with models that included age, one third (24, 32.0%) were categorical variables (Fig. 3), none of which had the exact same age levels. While most (68, 86.1%) of the 79 articles adjusting for smoking included smoking as a categorical variable, only 20 (20 of 68, 29.4%) were measured the exact same way (i.e., never smoking, former smoking, and current smoking). Of the 24 (24 of 65, 36.9%) models that included a measure of BMI as a categorical variable, eight (8 of 24, 33.3%) had cut-off levels that were used in at least one other study (*n* = 2 articles with < 20.0, 20.0–24.9, 25.0–29.9, 30.0–34.9, 35.0+ kg/m^2^ and *n* = 6 articles with < 25, 25–29.9, 30+ kg/m^2^).

### Confounding statements and bias considerations

Across all 87 articles, 56 (64.4%) included a specific mention of confounding bias in their Abstract and/or Discussion sections (Table 3). While another 18 (20.7%) articles alluded to the concept of confounding, without using any specific terminology, 13 (14.9%) did not mention or allude to confounding in their Abstract and/or Discussion sections. Over half (50, 57.5%) of the articles used the term “bias”. Among the eight mentions of bias that were related to the principle of confounding, three specifically included the words “confounding” or “confound”.

**Table 3:** Statements of confounding in studies assessing the impact of alcohol on ischemic heart disease

Question	
	No. (%, 95 Confidence Interval)
Total	87 (100)
Term “Confounding” mentioned in Abstract or Discussion	
Specific	56 (64.4, 54.0–74.7)
Alluded	18 (20.7, 12.6–29.9)
No	13 (14.9, 8.0–23.0)
Term “Bias” used in Abstract or Discussion	
Yes	50 (57.5, 47.1–67.8)
No	37 (42.5, 32.2–52.9)
Specific mention of non-adjusted confounders	
Yes	26 (29.9, 20.7–40.2)
*Not measured*	16 (61.5 42.3–80.8)
*Other reasons*	5 (19.2, 3.8–34.6)
*No reasons*	5 (19.2, 3.8–34.6)
No	61 (70.1, 59.8–79.3)
Any mention that findings may be affected by confounding?	
Likely	1 (1.2, 0.0–3.4)
Possibly	28 (32.2, 23.0–42.5)
Unlikely	15 (17.2, 9.2–25.3)
No statement	43 (49.4 39.1–59.8)
Cautious interpretation needed	
Yes	5 (5.7, 1.1–11.5)
No statement	82 (94.3, 88.5–98.9)
Conclusions include any limitations regarding confounding	
Yes	9 (10.3, 4.6–17.2)
No	78 (89.7, 82.8–95.4)

Nearly one-third (26, 29.9%) of the articles included a discussion regarding potential confounders for which there was no adjustment, and authors frequently (16 of 26, 61.5%) stated that these confounders had not been measured (Table 3).

Only one article specifically stated that their main findings were likely to be affected by residual confounding. Another 28 (32.2%) reported it was possible and 15 (17.2%) reported it was unlikely that their main findings were to be affected by residual confounding. There were five (5.7%) that explicitly asked for caution when interpreting results (Table 3).

Articles published after 2010 were more likely to include a specific mention of confounding (12 of 13, 92.3%), use the term “bias” (11 of 13, 84.6%), and ask for caution when interpreting results (2 of 13, 15.4%) (Additional file 5: Table S2).

## Discussion

Our analysis suggests that there is substantial variation in how adjustment, stratification, and matching confounders are defined, operationalized, and discussed across observational studies evaluating the impact of alcohol consumption on the risk of ischemic heart disease. While the majority of articles accounted for smoking, age, and BMI, these variables were rarely measured the exactly same way, and no two models considered the same higher-level confounder domains. Two-thirds of the articles specifically mentioned confounding bias in their Abstract and/or Discussion sections, but less than 2% claimed that their main findings were likely to be affected by residual confounding. Very few articles called for cautious interpretation due to confounding. Given the lack of standardized approaches for selecting and adjusting for confounders, and the inadequate discussions regarding the importance of confounding, individual findings from observational studies assessing the impact of alcohol consumption on ischemic heart disease may need to be interpreted with caution.

Most of the largest multivariate models in observational studies evaluating the impact of alcohol on the risk of ischemic heart disease accounted for smoking, age, BMI, and physical activity. Age and smoking are two of the most important risk factors for ischemic heart disease, and studies have regularly found that these variables confound the alcohol-ischemic heart disease relationship [21]. Although evidence on the influence of physical activity and BMI are sparse [21], a previous evaluation suggested that low BMI and leisure-time physical activity are more common among never-drinkers than among light drinkers [28]. However, the authors noted that the differences were unlikely to be large enough to explain the lower risk observed among light drinkers compared to abstainers [28]. Numerous studies have also indicated that drinking pattern and type of beverage are important confounders [29]. However, we only identified three studies evaluating drinking history in their largest multivariate models. Furthermore, other proposed potential confounders, including cognitive function, dietary habits, and socioeconomic status [12, 13, 30], were rarely evaluated. Overall, it is unclear whether the lack of consistency across articles reflects the fact that there is little consensus about which variables are potential confounders or the beliefs that the protective effect of alcohol consumption on the risk for cardiovascular disease is independent of how well studies control for confounding [13, 29, 31, 32].

We also found that articles rarely measured similar adjustment, matching, or stratification variables the same way. For instance, although standard categories for BMI have been proposed in the literature, only one-third of the categorical BMI variables in our sample had the same cut-off levels as at least one other study. These findings build upon previous concerns that it is often difficult to determine how categorical or continuous adjustment variables are treated in analyses [33, 34]. Moreover, different treatment of variables, including incorrect adjustment for continuous confounders, can have an impact on the observed estimates or result in residual confounding [35].

There are a number of reasons that could explain the variability in the adjustment, stratification, and matching variables. Although it is possible that authors may not be able to measure all potential confounders, and therefore are prevented from considering them in multivariate models, it is more likely that there is a lack of consensus about what should be considered as matching, adjustment, or stratification variables. Furthermore, researchers may not be reviewing previously published models to determine potentially important confounders. Different studies may have different rigor in measuring some variables, and this can affect whether investigators want to use these variables in their analyses. It is also possible that certain models are preferentially reported or excluded due to biases and potential conflicts of interest, especially if the unreported multivariate models resulted in less desirable results [27]. However, the optimal choice of covariates may be difficult to identify and consensus may be elusive even with the best intentions. While adjusting for a large number of potential confounders is often appropriate and necessary, it can be particularly challenging to differentiate between potential confounders and variables that may be in the path that explains the effect of a risk factor, which should not be adjusted for. Field-wide systematic exposure assessments may help standardize variable adjustments and identify the full range of potential effect estimates due to different modeling considerations (i.e., vibration of effects) [36]. Small changes in modelling choices, including exposure and outcome definitions, covariates considered, and statistical methods, can have a major impact on effect estimates observed in observational studies, and can even flip the direction of effects [36]. Furthermore, greater transparency when it comes to the choice, measurement, and impact of potential confounding variables is necessary. Without these efforts, the associations reported in observational studies of alcohol consumption on ischemic heart disease may need to be interpreted with great caution.

Our findings, which suggest that meta-analyses of observational studies evaluating the impact of alcohol consumption on the risk of ischemic heart disease are unlikely to identify effect estimates that have been adjusted for the same variables, are generalizable to other fields. When it comes to performing meta-analyses of observational studies, there are no clear rules regarding the prioritization of adjusted or unadjusted effect estimates. According to the Cochrane Handbook for Systematic Reviews of Interventions, review authors should record both adjusted and unadjusted effect estimates, but “no general recommendation can be made for the selection of which adjustment estimate is preferable” [37]. Instead, review authors are advised to consider the estimates from the models adjusted for the maximum number of covariates, the estimates from the primary models, or the estimates from the models with the largest number of confounders that are identified as important. Previous evaluations suggests that meta-analyses of observational studies evaluating the relationships between type 2 diabetes and cancer and environmental risk factors and dementia often only identify effect estimates from models that consider age and sex, despite a large number of other measurable confounders [38, 39]. Other assessments indicate a lack of consistency among the adjustment variables considered across individuals studies included in meta-analyses [40–42]. To facilitate the inclusion of effect estimates in meta-analyses, the raw data of individual studies should be made available to review authors in order to generate effect estimates across studies using the same or similar confounders. However, raw data are currently rarely available for observational studies [23, 25]. Even if they were available, it is likely that different datasets may vary substantially in what variables they have recorded.

We found that most authors mentioned the concept of confounding, which is consistent with prior evaluations of considerations of confounding in epidemiological studies [26]. However, we found that authors rarely explicitly state that their main findings should be interpreted with caution due to confounding. This is significantly lower than what has been previously observed among surveyed samples of high-impact observational studies and research focusing on medical interventions [34, 43]. Moving forward, more transparent reporting and discussions regarding the selection of confounders, including the potential impact of residual confounding, are necessary to ensure that observational associations on the relationship between alcohol consumption and ischemic heart disease can be properly interpreted.

This study has a number of potential limitations. First, our sample included 87 observational studies identified by a previous meta-analysis. Therefore, some articles may have been missed and the results may not be generalizable to all observational studies focusing on alcohol-health related outcomes. Although there may have been multiple reports of the same study, different authors evaluating the same data sources could have considered the same or different variables. Second, our study includes articles published between 1981 and 2015, and reporting practices may or may not have changed over time [43]. Our post-hoc analysis suggests that articles published after 2010 were more likely to mention confounding and biases. While these findings may suggest improvements over time, our sample was not designed to assess trends. Third, for each article, we focused only on the covariates included in the largest model. However, it is possible that authors may have considered additional potential confounders that were not eventually included in the largest model. Moreover, some variables included in the largest models may have been considered as predictors worth capturing, instead of potential confounders. However, it is difficult to assess which variables were explicitly deemed to be potential confounders. Fourth, given the different patient populations, it also may not have made sense to adjust for the same characteristics in all studies (e.g., adjusting for sex in a study of only women). This is why we reported separate results for studies that included both genders, only men, and only women. It is possible that some additional confounders beyond gender were dealt with by restriction, i.e. by using eligibility criteria that restricted upfront the study population to have the same value for a potential confounder.

## Conclusion

Our evaluation shows that although most authors mention confounding bias when interpreting their study findings, they rarely call for results to be interpreted with caution. However, the high variation in how confounders were defined and handled suggests that the results and their interpretation in these studies may have been affected by definition and handling choices.

## Supplementary information

**Additional file 1: Table S1.** Eligible studies.

**Additional file 2.** The higher-level confounder domains considered in 85 observational studies on alcohol and ischemic heart disease risk. 

**Additional file 3.** The full “data microarray” illustrating the higher-level confounder domains considered in 85 observational studies on alcohol and ischemic heart disease risk. Domains are ordered based on how many times they were included in multivariate models. Colors represent whether domains were adjustment, stratification, or matching variables and how they were measured.

**Additional file 4.** The full “data microarray” illustrating the higher-level confounder domains considered in 85 observational studies on alcohol exposure and ischemic heart disease, stratified by the type of population considered. Domains are ordered based on how many times they were included in multivariate models. Colors represent whether domains were adjustment, stratification, or matching variables and how they were measured.

**Additional file 5: Table S2.** Statements of confounding in studies assessing the impact of alcohol on ischemic heart disease by publication year.

## Data Availability

Data will be shared on https://osf.io/zrdx7/ upon publication.

## Abbreviations

BMI: Body mass index
GBD: Global Burden or Disease
