# Supplementary material for: Evaluation of confounding in epidemiologic studies assessing alcohol consumption on the risk of ischemic heart disease
Source: BMC Med Res Methodol. 2020 Mar 14;20:64. doi: 10.1186/s12874-020-0914-6 (PMC7071725; doi:10.1186/s12874-020-0914-6)
Supplement: Supplementary file 1 — Additional file 1: Table S1. Eligible studies. [file 12874_2020_914_MOESM1_ESM.docx]

| **Table S1. Eligible studies** | | | | |
| --- | --- | --- | --- | --- |
|  | **Area** | **Title** | **Year** | **Journal** |
| 1 | Ischemic heart disease | **"Moderate alcohol consumption and the risk of sudden cardiac death among US male physicians"** | 1999 | Circulation |
| 2 | Ischemic heart disease | **"Alcohol intake and the risk of coronary heart disease in the Spanish EPIC cohort study"** | 2012 | Heart |
| 3 | Ischemic heart disease | **"Alcohol consumption and risk for stroke among Chinese men"** | 2007 | Ann Neurol |
| 4 | Ischemic heart disease | **"The association of pattern of lifetime alcohol use and cause of death in the European prospective investigation into cancer and nutrition (EPIC) study"** | 2013 | Epidemiology |
| 5 | Ischemic heart disease | **"Alcohol consumption and the risk of acute myocardial infarction in women. "** | 1993 | J Epidemiol Community Health |
| 6 | Ischemic heart disease | **"Alcohol, drinking pattern and all-cause, cardiovascular and alcohol-related mortality in Eastern Europe"** | 2016 | Eur J Epidemiol |
| 7 | Ischemic heart disease | **"Alcohol drinking and mortality among men enrolled in an American Cancer Society prospective study** | 1990 | Epidemiology |
| 8 | Ischemic heart disease | **"Light-to-moderate alcohol consumption and risk of sudden cardiac death in women"** | 2010 | Heart Rhythm |
| 9 | Ischemic heart disease | **"Moderate alcohol and decreased cardiovascular mortality in an elderly cohort"** | 1985 | Am Heart J |
| 10 | Ischemic heart disease | **"Alcohol and mortality in Busselton, Western Australia"** | 1993 | Am J Epidemiol |
| 11 | Ischemic heart disease | **"Higher usual alcohol consumption was associated with a lower 41-y mortality risk from coronary artery disease in men independent of genetic and common environmental factors: the prospective NHLBI Twin Study"** | 2015 | Am J Clin Nutr |
| 12 | Ischemic heart disease | **"Alcohol consumption and risk of cardiovascular disease and death in women: potential mediating mechanisms"** | 2009 | Circulation |
| 13 | Ischemic heart disease | **"Mortality in relation to alcohol consumption: a prospective study among male British doctors."** | 2005 | Int J Epidemiol |
| 14 | Ischemic heart disease | **"Alcohol drinking pattern and non-fatal myocardial infarction in women"** | 2007 | Addiction |
| 15 | Ischemic heart disease | **"The association of alcohol consumption with coronary heart disease mortality and cancer incidence varies by smoking history"** | 2005 | J Gen Intern Med |
| 16 | Ischemic heart disease | **"Type of alcoholic beverage and first acute myocardial infarction: a case-control study in a Mediterranean country"** | 2003 | Clin Cardio |
| 17 | Ischemic heart disease | **"Coronary heart disease mortality and alcohol consumption in Framingham"** | 1986 | Am J Epidemiol |
| 18 | Ischemic heart disease | **"Alcohol consumption and mortlity among women"** | 1995 | NEJM |
| 19 | Ischemic heart disease | **"Association between alcoholic beverage consumption and incidence of coronary heart disease in whites and blacks: the Atherosclerosis Risk in Communities Study"** | 2004 | Am J Epidemiol |
| 20 | Ischemic heart disease | "Alcohol consumption and risk of ischemic heart disease in women" | 1993 | Arch Intern Med |
| 21 | Ischemic heart disease | **"Light-to-moderate alcohol consumption and mortality in the Physicians' Health Study enrollment cohort"** | 2000 | J Am Coll Cardiol |
| 22 | Ischemic heart disease | **"Does alcohol protect against ischaemic heart disease in Bulgaria? A case-control study of non-fatal myocardial infarction in Sofia."** | 2001 | Cent Eur J Public Health |
| 23 | Ischemic heart disease | **"A prospective study of the health effects of alcohol consumption in middle-aged and elderly men. The Honolulu Heart Program"** | 1994 | Circulation |
| 24 | Ischemic heart disease | **"Alcohol consumption and risk of heart failure: the Atherosclerosis Risk in Communities Study"** | 2015 | Eur Heart J |
| 25 | Ischemic heart disease | **"Drinking and coronary heart disease: the Albany Study"** | 1985 | Am Heart J |
| 26 | Ischemic heart disease | **"Type of alcohol consumed and mortality from all causes, coronary heart disease, and cancer"** | 2000 | Ann Intern Med |
| 27 | Ischemic heart disease | **"Alcohol consumption, drinking pattern and acute myocardial infarction. A case referent study based on the Swedish Twin Register"** | 1997 | J Intern Med |
| 28 | Ischemic heart disease | **"Alcohol consumption and cardiovascular mortality accounting for possible misclassification of intake: 11-year follow-up of the Melbourne Collaborative Cohort Study"** | 2007 | Addiction |
| 29 | Ischemic heart disease | **"Alcohol consumption and mortality and hospital admissions in men from the Midspan collaborative cohort study"** | 2008 | Addiction |
| 30 | Ischemic heart disease | **"Alcohol consumption, Lewis phenotypes, and risk of ischaemic heart disease."** | 1993 | Lancet |
| 31 | Ischemic heart disease | **"Established risk factors account for most of the racial differences in cardiovascular disease mortality"** | 2007 | PLOS One |
| 32 | Ischemic heart disease | **"Genetic variation in alcohol dehydrogenase and the beneficial effect of moderate alcohol consumption on myocardial infarction"** | 2001 | NEJM |
| 33 | Ischemic heart disease | **"Familial predisposition and susceptibility to the effect of other risk factors for myocardial infarction."** | 1999 | J Epidemiol Community Health |
| 34 | Ischemic heart disease | **"Alcohol consumption and mortality from stroke and coronary heart disease among Japanese men and women: the Japan collaborative cohort study"** | 2008 | Stroke |
| 35 | Ischemic heart disease | **"Alcohol consumption, social support, and risk of stroke and coronary heart disease among Japanese men: the JPHC Study"** | 2009 | Alcohol Clin Exp Res |
| 36 | Ischemic heart disease | **"Alcohol intake and the risk of cardiovascular disease in middle-aged Japanese men"** | 1995 | Stroke |
| 37 | Ischemic heart disease | **". Alcohol consumption and risk of coronary heart disease"** | 1991 | BMJ |
| 38 | Ischemic heart disease | **"Alcohol consumption and mortality in Serbia: twenty-year follow-up study. "** | 2004 | Croat Med J |
| 39 | Ischemic heart disease | **"Alcohol intake, drinking patterns, and risk of nonfatal acute myocardial infarction in Costa Rica"** | 2005 | Am J Clin Nutr |
| 40 | Ischemic heart disease | **"Alcohol and cardiovascular disease: the Hawaiian experience."** | 1981 | Circulation |
| 41 | Ischemic heart disease | **" A case-control study of coronary heart disease in Athens, Greece"** | 1992 | Int J Epidemiol |
| 42 | Ischemic heart disease | **"Alcoholic beverages and myocardial infarction in young men"** | 1985 | Am J Epidemiol |
| 43 | Ischemic heart disease | **"Mortality in British vegetarians: results from the European Prospective Investigation into Cancer and Nutrition (EPIC-Oxford)."** | 2009 | Am J Clin Nutr |
| 44 | Ischemic heart disease | **"Alcohol intake and premature coronary heart disease in urban Japanese men"** | 1998 | Am J Epidemiol |
| 45 | Ischemic heart disease | **"Alcohol consumption and mortality in aging or aged Finnish men"** | 1989 | J Clin Epidemiol |
| 46 | Ischemic heart disease | **"Alcohol intake and nonfatal acute myocardial infarction in Japan."** | 1991 | Am J Cardiol |
| 47 | Ischemic heart disease | **"Alcohol and mortality: a cohort study of male japanese physicians** | 1986 | Int J Epidemiol |
| 48 | Ischemic heart disease | **"Change in alcohol consumption and risk of death from all causes and from ischaemic heart disease."** | 1991 | BMJ |
| 49 | Ischemic heart disease | **" Alcohol intake and risk of acute coronary syndrome and mortality in men and women with and without hypertension"** | 2011 | Eur J Epidemiol |
| 50 | Ischemic heart disease | **"Heavy and nonheavy drinking occasions, all-cause and cardiovascular mortality and hospitalizations: a follow-up study in a population with a low consumption level."** | 2005 | J Stud Alcohol |
| 51 | Ischemic heart disease | **"Relation between heavy and binge drinking and all-cause and cardiovascular mortality in Novosibirsk, Russia: a prospective cohort study"** | 2002 | Lancet |
| 52 | Ischemic heart disease | **"Alcohol consumption and cardiovascular disease: differential effects in France and Northern Ireland"** | 2004 | Eur J Cardiovasc |
| 53 | Ischemic heart disease | **"Alcohol consumption: protection against coronary heart disease and risks to health."** | 1990 | Int J Epidemiol |
| 54 | Ischemic heart disease | **"Roles of drinking pattern and type of alcohol consumed in coronary heart disease in men"** | 2003 | NEJM |
| 55 | Ischemic heart disease | **"Alcohol volume, drinking pattern, and cardiovascular disease morbidity and mortality: is there a U-shaped function? "** | 2001 | Am J epidemiol |
| 56 | Ischemic heart disease | **"The combined influence of leisure-time physical activity and weekly alcohol intake on fatal ischaemic heart disease and all-cause mortality"** | 2008 | Eur Heart J |
| 57 | Ischemic heart disease | **"Central adiposity and increased risk of coronary artery disease mortality in older women"** | 1993 | Ann Epidemiol |
| 58 | Ischemic heart disease | **"Alcohol consumption and coronary heart disease morbidity and mortality"** | 1997 | Am J Epidemiol |
| 59 | Ischemic heart disease | **"Alcohol and mortality in middle-aged men from eastern France. "** | 1998 | Epidemiology |
| 60 | Ischemic heart disease | **"Prospective study of alcohol consumption and risk of coronary disease in men"** | 1991 | Lancet |
| 61 | Ischemic heart disease | **"Heavy drinking occasions in relation to ischaemic heart disease mortality– an 11-22 year follow-up of the 1984 and 1995 US National Alcohol Surveys."** | 2011 | Int J Epidemiol |
| 62 | Ischemic heart disease | **"Alcohol, mortality and cardiovascular events in a 35 year follow-up of a nationwide representative cohort of 50,000 Swedish conscripts up to age 55"** | 2012 | Alcohol and Alcoholism |
| 63 | ischemic heart disease | **"Abstention, alcohol use and risk of myocardial infarction in men and women taking account of social support and working conditions: the SHEEP case-control study"** | 2012 | Alcohol and Alcoholism |
| 64 | Ischemic heart disease | **"Association between alcohol consumption and mortality, myocardial infarction, and stroke in 25 year follow up of 49 618 young Swedish men"** | 1999 | BMJ |
| 65 | Ischemic heart disease | **"Alcohol consumption and mortality risks in the USA"** | 2012 | Alcohol and Alcoholism |
| 66 | Ischemic heart disease | **"Myocardial infarction and alcohol consumption: a population-based case-control study"** | 2007 | Nutr Metab Cardiovasc Dis |
| 67 | Ischemic heart disease | **"Alcohol and exercise in myocardial infarction and sudden coronary death in men and women."** | 1987 | Am J Epidemiol |
| 68 | Ischemic heart disease | **"No Protective Effect of Alcohol Consumption on Coronary Heart Disease (CHD) in African Americans: Average Volume of Drinking over the Life Course and CHD Morbidity and Mortality in a U.S. National Cohort"** | 2002 | Contemporary Drug Problems |
| 69 | Ischemic heart disease | **"Alcohol intake and subsequent mortality: findings from the NHANES I Follow-up Study"** | 1995 | J Stud Alcohol |
| 70 | Ischemic heart disease | **"Alcohol and coronary heart disease: a perspective from the British Regional Heart Study"** | 1994 | Int J Epidemiol |
| 71 | Ischemic heart disease | **"Alcohol intake and survival in the elderly: a 77 month follow-up in the Dubbo study"** | 1996 | Aust NZ J Med |
| 72 | Ischemic heart disease | **"A prospective study of moderate alcohol consumption and the risk of coronary disease and stroke in women"** | 1988 | NEJM |
| 73 | Ischemic heart disease | **"Long-term wine consumption is related to cardiovascular mortality and life expectancy independently of moderate alcohol intake: the Zutphen Study"** | 2009 | J Epidemiol Community Health |
| 74 | Ischemic heart disease | **"Alcohol use and mortality from coronary heart disease: the role of high-density lipoprotein cholesterol. The Multiple Risk Factor Intervention Trial Research Group"** | 1992 | Ann Intern Med |
| 75 | Ischemic heart disease | **"Alcohol consumption and sudden coronary death in middle-aged Finnish men"** | 1987 | J intern Med |
| 76 | Ischemic heart disease | "Risk factors for non-fatal acute myocardial infarction in Italian women" | 2004 | Prev Med |
| 77 | Ischemic heart disease | **"Alcohol consumption and mortality among middle-aged and elderly U.S. adults. "** | 1997 | NEJM |
| 78 | Ischemic heart disease | **"Alcohol and sudden cardiac death."** | 1992 | Br Heart J |
| 79 | Ischemic heart disease | **"Alcohol consumption and its contribution to the burden of coronary heart disease in middle-aged and older New Zealanders: a population-based case-control study"** | 2004 | N Z Med J |
| 80 | Ischemic heart disease | **"Moderate alcohol consumption and heart disease."** | 2002 | Health Rep |
| 81 | Ischemic heart disease | **" Joint effect of cigarette smoking and alcohol consumption on mortality"** | 2007 | Prev Med |
| 82 | Ischemic heart disease | **"Alcohol drinking and overall and cause-specific mortality in China: nationally representative prospective study of 220,000 men with 15 years of follow-up"** | 2012 | Int J Epidemiol |
| 83 | Ischemic heart disease | **"Genetic variation in alcohol dehydrogenase 1C and the beneficial effect of alcohol intake on coronary heart disease risk in the Second Northwick Park Heart Study"** | 2005 | Atherosclerosis |
| 84 | Ischemic heart disease | **" Follow up study of moderate alcohol intake and mortality among middle aged men in Shanghai, China"** | 1997 | BMJ |
| 85 | Ischemic heart disease | **"Alcohol consumption and mortality in an American male population: recovering the U-shaped curve–findings from the normative Aging Study"** | 1992 | J Stud Alcohol |
| 86 | Ischemic heart disease | **"Alcohol consumption, genetic variants in alcohol deydrogenases, and risk of cardiovascular diseases: a prospective study and meta-analysis"** | 2012 | PLOS One |
| 87 | Ischemic heart disease | **"Alcohol and mortality"** | 1992 | Ann Intern Med |
