# Supplementary figures and images for: Evaluation of confounding in epidemiologic studies assessing alcohol consumption on the risk of ischemic heart disease
Source: BMC Med Res Methodol. 2020 Mar 14;20:64. doi: 10.1186/s12874-020-0914-6 (PMC7071725; doi:10.1186/s12874-020-0914-6)

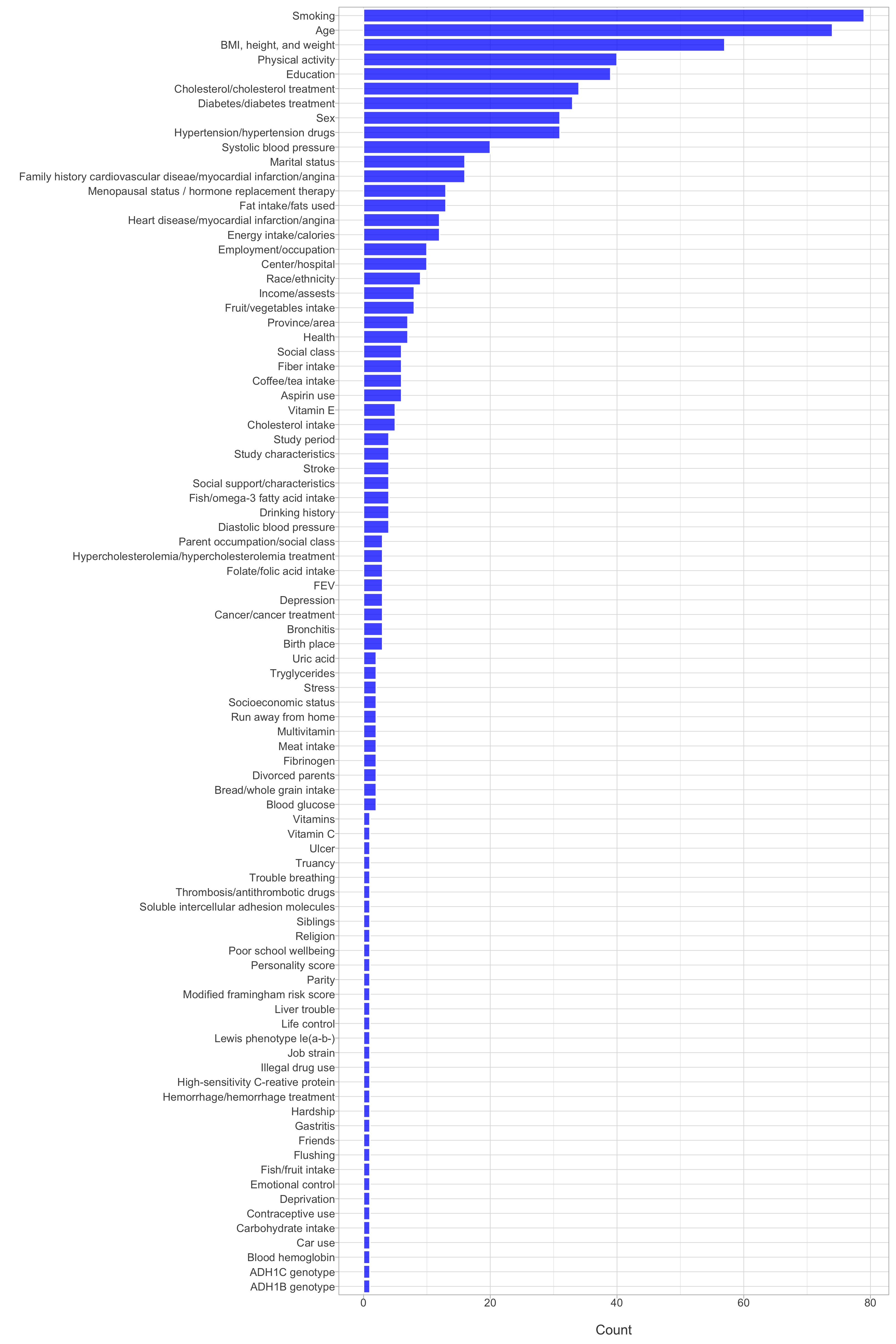

Supplement: Supplementary file 2 — Additional file 2. The higher-level confounder domains considered in 85 observational studies on alcohol and ischemic heart disease risk. [file 12874_2020_914_MOESM2_ESM.jpg]

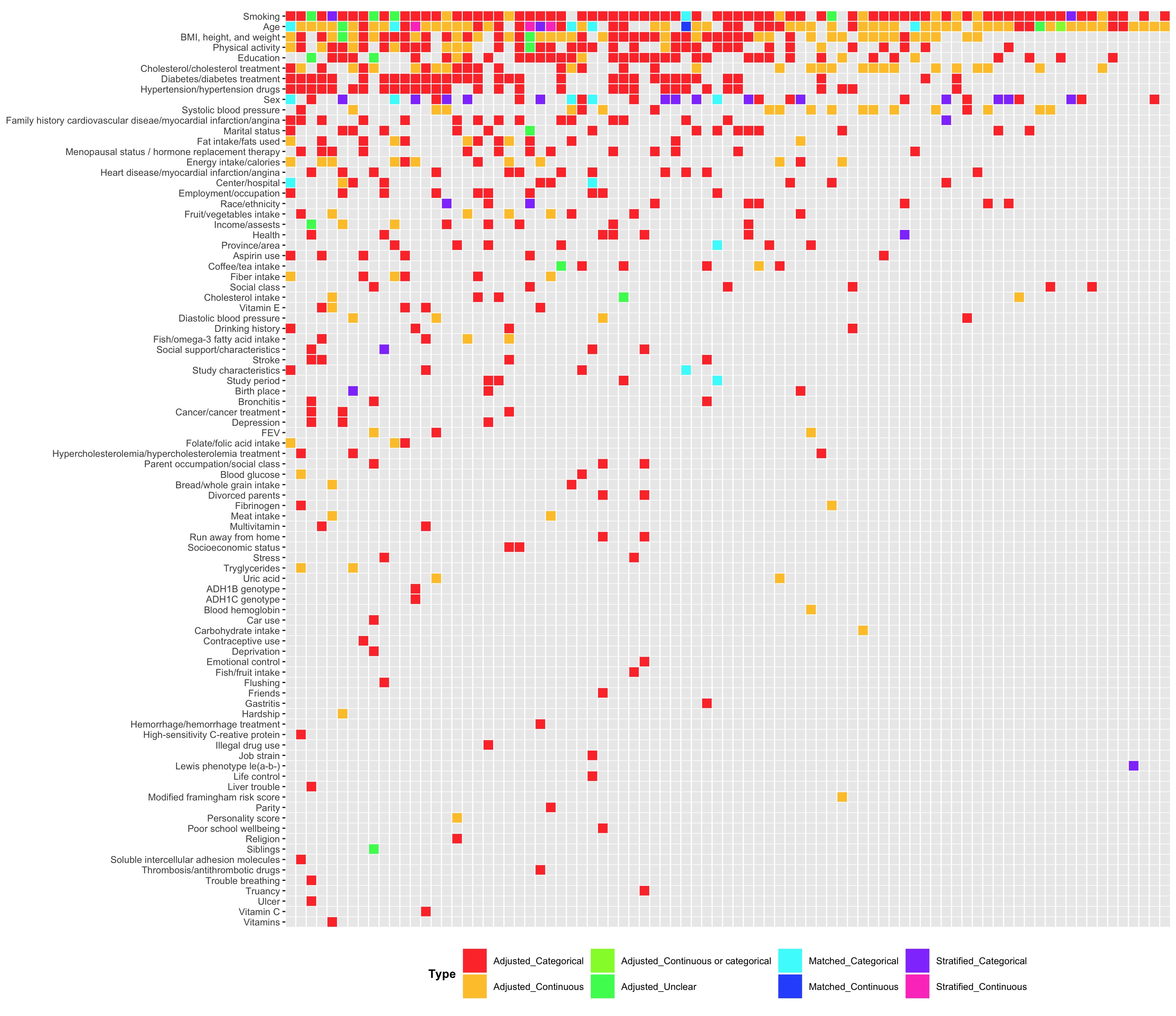

Supplement: Supplementary file 3 — Additional file 3. The full “data microarray” illustrating the higher-level confounder domains considered in 85 observational studies on alcohol and ischemic heart disease risk. Domains are ordered based on how many times they were included in multivariate models. Colors represent whether domains were adjustment, stratification, or matching variables and how they were measured. [file 12874_2020_914_MOESM3_ESM.jpg]

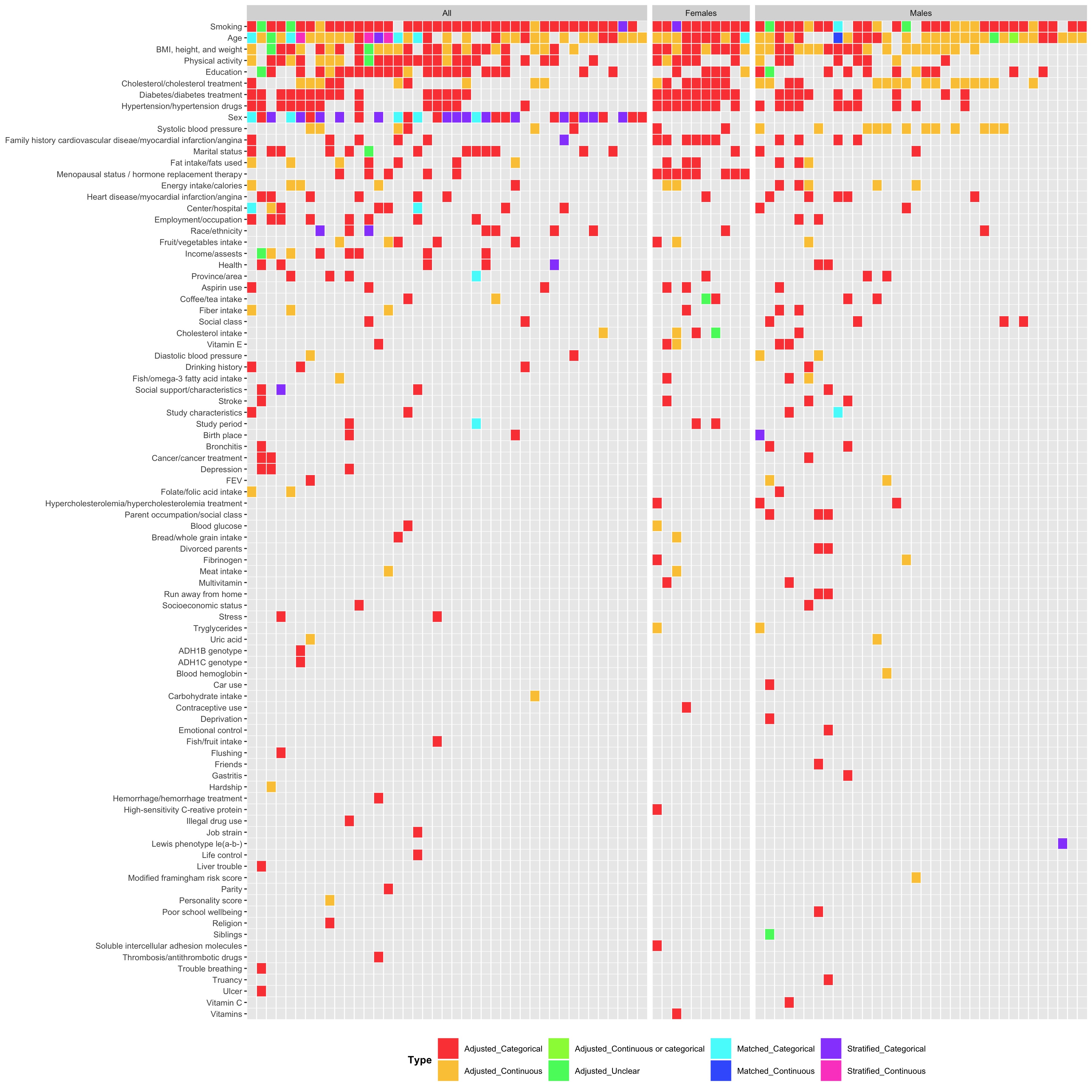

Supplement: Supplementary file 4 — Additional file 4. The full “data microarray” illustrating the higher-level confounder domains considered in 85 observational studies on alcohol exposure and ischemic heart disease, stratified by the type of population considered. Domains are ordered based on how many times they were included in multivariate models. Colors represent whether domains were adjustment, stratification, or matching variables and how they were measured. [file 12874_2020_914_MOESM4_ESM.jpg]
