# Supplementary material for: Evaluation of confounding in epidemiologic studies assessing alcohol consumption on the risk of ischemic heart disease
Source: BMC Med Res Methodol. 2020 Mar 14;20:64. doi: 10.1186/s12874-020-0914-6 (PMC7071725; doi:10.1186/s12874-020-0914-6)
Supplement: Supplementary file 5 — Additional file 5: Table S2. Statements of confounding in studies assessing the impact of alcohol on ischemic heart disease by publication year. [file 12874_2020_914_MOESM5_ESM.docx]

| **Table S2. Statements of confounding in studies assessing the impact of alcohol on ischemic heart disease by publication year** | | | | | | |
| --- | --- | --- | --- | --- | --- | --- |
| **Question** | | **N = 10** | **N = 31** | **N = 33** | **N = 13** | **No. (%, 95% Confidence Interval)** |
| Total |  | <1999 | 1990-1999 | 2000-2009 | 2010+ | Total |
| Term “Confounding” mentioned in Abstract or Discussion |  |  |  |  |  |  |
|  | Specific | 6 (60.0) | 15 (48.4) | 23 (69.7) | 12 (92.3) | 56 (64.4) |
|  | Alluded | 4 (40.0) | 7 (22.6) | 6 (18.2) | 1 (7.7) | 18 (20.7) |
|  | No | 0 (0.0) | 9 (29.0) | 4 (12.1) | 0 (0.0) | 13 (14.9) |
| Term “Bias” used in Abstract or Discussion |  |  |  |  |  |  |
|  | Yes | 5 (50.0) | 16 (51.6) | 18 (54.5) | 11 (84.6) | 50 (57.5) |
|  | No | 5 (50.0) | 15 (48.4) | 15 (45.5) | 2 (15.4) | 37 (42.5) |
| Specific mention of non-adjusted confounders |  |  |  |  |  |  |
|  | Yes | 1 (10.0) | 4 (12.9) | 17 (51.5) | 4 (30.8) | 26 (29.9) |
|  | No | 9 (90.0) | 27 (87.1) | 16 (48.5) | 9 (69.2) | 61 (70.1) |
| Any mention that findings may be affected by confounding? |  |  |  |  |  |  |
|  | Likely | 0 (0.0) | 1 (3.2) | 0 (0.0) | 0 (0.0) | 1 (1.2) |
|  | Possibly | 2 (20.0) | 4 (12.9) | 16 (48.5) | 6 (46.2) | 28 (32.2) |
|  | Unlikely | 2 (20.0) | 3 (9.7) | 4 (12.1) | 6 (46.2) | 15 (17.2) |
|  | No statement | 6 (60.0) | 23 (74.2) | 13 (39.4) | 1 (7.7) | 43 (49.2) |
| Cautious interpretation needed |  |  |  |  |  |  |
|  | Yes | 1 (10.0) | 1 (3.2) | 1 (3.0) | 2 (15.4) | 5 (5.7) |
|  | No statement | 9 (10.0) | 30 (96.8) | 32 (97.0) | 11 (84.6) | 82 (94.3) |
| Conclusions include any limitations regarding confounding |  |  |  |  |  |  |
|  | Yes | 1 (10.0) | 3 (9.7) | 2 (6.1) | 3 (23.1) | 9 (10.3) |
|  | No | 9 (90.0) | 28 (90.3) | 31 (93.9) | 10 (76.9) | 78 (89.7) |
